# Impact of Simulated Microgravity on Oligodendrocyte Development: Implications for Central Nervous System Repair

**DOI:** 10.1371/journal.pone.0076963

**Published:** 2013-12-04

**Authors:** Araceli Espinosa-Jeffrey, Pablo M. Paez, Veronica T. Cheli, Vilma Spreuer, Ina Wanner, Jean de Vellis

**Affiliations:** 1 Semel Institute for Neuroscience and Human Behavior, David Geffen School of Medicine at UCLA, Intellectual and Developmental Disabilities Research Center, Los Angeles, California, United States of America; 2 Hunter James Kelly Research Institute, Department of Pharmacology and Toxicology, School of Medicine and Biomedical Sciences, SUNY at Buffalo, NYS Center of Excellence, Buffalo, New York, United States of America; Universidade Federal do Rio de Janeiro, Brazil

## Abstract

We have recently established a culture system to study the impact of simulated microgravity on oligodendrocyte progenitor cells (**OPCs**) development. We subjected mouse and human OPCs to a short exposure of simulated microgravity produced by a 3D-Clinostat robot. Our results demonstrate that rodent and human OPCs display enhanced and sustained proliferation when exposed to simulated microgravity as assessed by several parameters, including a decrease in the cell cycle time. Additionally, OPC migration was examined *in vitro* using time-lapse imaging of cultured OPCs. Our results indicated that OPCs migrate to a greater extent after stimulated microgravity than in normal conditions, and this enhanced motility was associated with OPC morphological changes. The lack of normal gravity resulted in a significant increase in the migration speed of mouse and human OPCs and we found that the average leading process in migrating bipolar OPCs was significantly longer in microgravity treated cells than in controls, demonstrating that during OPC migration the lack of gravity promotes leading process extension, an essential step in the process of OPC migration. Finally, we tested the effect of simulated microgravity on OPC differentiation. Our data showed that the expression of mature oligodendrocyte markers was significantly delayed in microgravity treated OPCs. Under conditions where OPCs were allowed to progress in the lineage, simulated microgravity decreased the proportion of cells that expressed mature markers, such as CC1 and MBP, with a concomitant increased number of cells that retained immature oligodendrocyte markers such as Sox2 and NG2. Development of methodologies aimed at enhancing the number of OPCs and their ability to progress on the oligodendrocyte lineage is of great value for treatment of demyelinating disorders. To our knowledge, this is the first report on the gravitational modulation of oligodendrocyte intrinsic plasticity to increase their progenies.

## Introduction

Myelin is essential for the efficient conduction of electrochemical messages to and from the central nervous system (**CNS**) and lack of myelin due to trauma and/or disease leads to CNS dysfunction. White matter disorders encompass a wide spectrum of medical conditions that can occur prenatally and extend into adulthood and old age. In many cases OPCs fail to mature and therefore, they cannot remyelinate axons in dys- and demyelinating diseases such as Multiple Sclerosis, Pelizaeus-merzbacher's disease or after a traumatic injury. Enhanced remyelination through transplantation of OPCs may offer a realistic approach to restoring meaningful neurological function. Yet, effective methods to generate OPCs that would be devoid of other cell populations prior to be grafted, still need to be developed.

Taking advantage of the intrinsic plasticity of OPCs we designed a completely different approach aiming at producing larger numbers of OPCs than with conventional methods. We combined the use of simulated microgravity (**0G**) and our proprietary culture media. We used the Mitsubishi 3D-clinostat (three dimensional clinostat) which has been developed for simulation of microgravity on earth. Microgravity has been applied in many disciplines in gravitational biology. Previous studies have shown that microgravity suppresses the differentiation of human hematopoietic progenitor cells [1] and human osteoblast cells [2]. Yuge and coworkers [3] reported that human mesenchymal stem cells cultured under 0G maintained their undifferentiated state, differentiated into hyaline cartilage after being transplanted into cartilage defective mice, and had a high survival rate. More recent work tested the effects of 0G on bone marrow stromal cells. Neural-induced mesenchymal bone marrow stromal cells cultured under 1G conditions exhibited neural differentiation, whereas those cultured under 0G did not. Moreover, when these cells were administered intra-venously into a mouse model of cerebral contusion, they survived in larger numbers than cells grown in 1G [4].

The goal of this work was to develop a method to obtain OPCs more rapidly and with a higher yield than is currently possible. Here, we report for the first time a gravitational modulation of oligodendrocyte development. We found that simulated microgravity promotes mouse and human OPC proliferation, motility and retain OPCs in an immature stage. Currently there are treatments for the symptoms resulting from myelin deficiency but, there is no cure for myelin disorders despite their increasing prevalence and clinical importance. Therefore, this work is relevant to myelin diseases as cell replacement therapies represent a promising approach to enhance and sustain remyelination.

## Methods

### Animals

The mice colony was housed in our animal facilities with a 12 h light, 12 h dark regime. 0–1 day old pups were used to prepare the mixed glial cultures. Cultures were performed in accordance with the NIH guidelines for the Care and Use of Laboratory Animals, and approved by the UCLA Chancellor's Animal Research Committee.

### Human cells

Human tissue experiments were approved by the Office of the Human Subject Committee. Cultures were prepared with fetal human tissue specimens donated by the Department of Pathology and Laboratory of Medicine at UCLA. Samples were de-identified in accordance with the NIH guidelines. These anonymous, pre-shelved specimens are donated for medical research purposes and are IRB exempt. (www.pathology.ucla.edu/TPCL.html).

### Preparation of mixed glial cultures

Mixed glial culture of GFP labeled OPCs were prepared as previously described [5], [6]. First, cerebral hemispheres from 1 day old mice were mechanically dissociated and were plated on poly-D-lysine-coated flasks in Dulbecco's modified Eagle's medium and Ham's F12 (1∶1 vol/vol) (Invitrogen Life Technologies), containing 100 µg/ml gentamycin and supplemented with 4 mg/ml dextrose anhydrous, 3.75 mg/ml HEPES buffer, 2.4 mg/ml sodium bicarbonate and 10% fetal bovine serum (FBS) (Omega Scientific). After 24 h the medium was changed and the cells were grown in DMEM/F-12 supplemented with insulin (5 µg/ml), human transferrin (50 µg/ml), sodium selenite (30 nM), d-Biotin (10 mM), 0.1% BSA (Sigma Aldrich), 1% horse serum and 1% FBS (Omega Scientific). After 9 days, the medium was changed and the mixed glial cultures were ready to use.

### Preparation of human embryonic brain derived OPCs

Human cortical tissue samples were de-identified donated voluntary aborted specimen remains pre-shelved in Pathology and thereby Institutional Review Board exempt. Neural progenitors were purified from a cell suspension of human fetal cortex at 15–17 weeks of gestation using a percoll gradient [7]. The volume of the filtered cell suspension in growth medium (DMEM/F12, 10% FBS, 1∶1000 gentamicin) was determined and a final 30% percoll solution was made by mixing one part of a HBSS-buffered percoll solution to two parts of cell suspension. The mixture was centrifuged in Oakridge tubes at 30,000 g at 4°C for 30 min. The bottom half fraction was transferred to a new 50 ml tube and the pellet of blood cells was discarded. Then, the percoll suspension was diluted in two volumes of growth medium and the cells were collected by centrifugation at 400 g for 10 min. The cell pellet was resuspended with 20 ml of growth medium per tube and plated onto uncoated 100 mm non adherent petri dishes. After 48 h, the cell suspension from two dishes was transferred into a 50 ml tube and centrifuged for 5 min at 400 g, next the cells were resuspend in 24 ml of fresh growth medium and plated in 75 mm tissue culture flasks (12 ml/flasks). The following day, 6 ml of growth medium was replaced with oligodendrocyte specification medium (**OSM**) to induce oligodendrocyte lineage commitment. This last step was repeated every four days until obtaining the desired cell density. After reaching confluency, cells were mechanically detached and replated in OSM either in 12.5 mm flasks or glass flaskettes. After 72 h half of the cultures were placed in the 3D-Clinostat and half cultures were kept in the same incubator in 1G.

We previously documented protocols for the production, isolation, and maintenance of the oligodendrocytes derived from rodent and human neural stem cells using chemically defined media [8], [9]. What makes this a unique system is a series of chemically defined media, specifically designed and carefully characterized for each developmental stage of oligodendrocytes as they advance from OPCs to mature, myelinating oligodendrocyte. For the purpose of the present study we used the oligodendrocyte specification medium (OSM). Our culture media formulation includes the minimum and sufficient nutrients to support a given developmental stage; thus, OPCs can be kept indefinitely under the specific conditions defined for the progenitor stage in OSM.

### Simulated microgravity (0G) induced by the 3D-Clinostat

Mixed glial culture of GFP labeled OPCs and cultures of human embryonic brain derived OPCs were grown during different periods of time in a 3D-Clinostat placed inside a CO2 incubator. The 3D-Clinostat, a multidirectional G force generator, was produced by Mitsubishi Heavy Industries. By controlled simultaneous rotation of 2 axes, the 3D-Clinostat cancels the cumulative gravity vector at the center around the device, producing an environment with an average of 10^−3^ G over time. This is accomplished by rotation of a chamber at the center of the device to disperse the gravity vector uniformly within a spherical volume, at a constant angular velocity. The outline of operational principle is described together with its mechanical design as follows: rotation around two independent axes makes the direction of gravity vector to scan whole steric angle. The magnitude and direction of rotational angular velocity is selected randomly at a certain interval of time to avoid singularity in sweep trajectory of gravity vector. The 3D-Clinostat is available in the Cell Biology Core of the Intellectual and Developmental Disabilities Research Center (IDDRC) at UCLA.

### Immunocytochemistry

Mixed glial culture of GFP labeled OPCs and cultures of human embryonic brain derived OPCs were stained with antibodies against neuron-glial antigen 2 (NG2), Sox transcription factor 2 (Sox2), Sox transcription factor 9 (Sox9), oligodendrocyte transcription factor 1 (Olig1), oligodendrocyte transcription factor 2 (Olig2), APC (CC1) and myelin basic protein (MBP) and examined by confocal microscopy. The cells were rinsed briefly in PBS and fixed in 4% buffered paraformaldehyde for 30 min at room temperature. After rinsing in PBS, the cells were permeabilized with 0.1% Triton X-100 in PBS for 10 min at room temperature and then processed for immunocytochemistry as described previously [10]. Essentially, fixed cells were incubated in a blocking solution (5% goat serum in PBS) followed by an overnight incubation at 4°C with the corresponding primary antibody. Staining with NG2 was performed on live cells without permeabilization for 1 h at room temperature before fixation. Cells were then incubated with the appropriate secondary antibodies (1∶200; Jackson ImmunoResearch), mounted onto slides with Aquamount (Lerner Laboratories), and fluorescent images were obtained using a Olympus spinning disc confocal microscope. Nuclei were stained with the fluorescent dye DAPI (5 µg/ml in 1% DMSO), in order to determine the total number of cells. Quantitative analysis of the results was done counting the antigen-positive and DAPI-positive cells in 20 randomly selected fields, which resulted in counts of >2,000 cells for each experimental condition. Counts of antigen-positive cells were normalized to the counts of total DAPI-positive cells for each condition. The primary antibodies used for immunohistochemistry were against: CC1 (1∶300; Calbiochem/EMD Biosciences) and MBP (1∶1000; Covance); NG2 (1∶400), Olig1 (1∶500), Olig2 (1∶500), Sox2 (1∶200) and Sox9 (1∶500) all from Millipore. Data are presented as mean ± SEM and statistical significance was assessed by using the Student paired t test, in which p<0.05 was defined as statistically significant.

### Incorporation of 5-bromo 2-deoxyuridine (BrdU)

Twenty four hour pulses of 10 µM bromo-deoxyuridine (BrdU) (BD Pharmingen) were applied at different time points in 0G treated and control cells. After each BrdU pulse, cells were fixed and immunostained in order to determine the number of positive cells. The cells were fixed in 4% paraformaldehyde in PBS. After treatment with 6 N HCl and 1% Triton X-100 to denature nuclear DNA, the cells were incubated in 0.1 M sodium borate (in PBS and 1% Triton X-100) for 10 min. Immunocytochemistry was done using an anti-BrdU antibody (1/1000; BD Pharmingen) and an anti-NG2 (1/100; Chemicon) with the corresponding fluorescent secondary antibodies. The percentage of BrdU positive cells was estimated on the basis of the total number of NG2 positive cells. Data are presented as mean ± SEM and statistical significance was assessed by using the Student paired t test, in which p<0.05 was defined as statistically significant.

### Cell cycle time analysis

To examine cell cycle time (Tc), mixed glial culture of GFP labeled OPCs [11] and cultures of human embryonic brain derived OPCs were used. These cultures were incubated in a stage top chamber with 5% CO2 at 37°C, which was placed on the stage of a spinning disc confocal inverted microscope equipped with a motorized stage, an atmosphere regulator, and shutter control. Fluorescent and bright-field images were obtained at 6 min intervals. Individual clones of mouse and human OPCs were followed for a period of 26 h. In these time-lapse experiments, ∼30 clones were analyzed per experimental condition.

SlideBookTM 4.1 (SlideBookTM 4.1, Intelligent Imaging Innovations) was used in the analysis of video-image sequences. The SlideBook software allow an investigator to cycle back-and-forth through the movie files frame-by-frame (minute-by-minute), facilitating the accurate determination of event time. To quantitatively analyze the dynamics of cell division, 120 cytokinetic events were randomly selected from movies at different time points. Cell proliferation was assessed by calculating the average cell cycle time (time between birth cytokinesis and division cytokinesis) in different OPC clones. Tracking of cells was performed by visual observation of image sequences as described above. In some cases the cells were semiautomatically followed in SlideBook by attaching a number to the cell, which was propagated from frame to frame. Tracking was performed forwards and backwards in time from each identified cytokinesis events to maximize the number of lineally related cytokineses identified. Furthermore, the percentage of cycling OPCs during the first 12 h of the time-lapse experiment was performed by visual observation. Data are presented as mean ± SEM and statistical significance was assessed by using the Student paired t test, in which p<0.05 was defined as statistically significant.

### Cell migration analysis by time-lapse

Immediately after removal from the 3-D Clinostat mouse OPCs or human embryonic brain derived OPCs were incubated in a stage top chamber with 5%CO2 at 37°C (Live-Cell Control Unit), which was placed on the stage of a Olympus spinning disc confocal inverted microscope equipped with a motorized z-stage. A 20× objective was used for acquiring images. Bright-field images were acquired for human OPCs, whereas fluorescent field images were obtained for mixed glial cultures of PLP-GFP OPCs with a specific GFP filter at 0.5 ms exposure times. Images were taken every 6 min over a period of 16 h using a CCD camera (Hamamatsu ORCA-R2) and a Image analysis software (SlideBookTM 4.1, Intelligent Imaging Innovations). Cell migration speed and distances were analyzed off-line by tracing individual cells using the motion tracking function of SlideBook software [12]. The brightest part of each cell body was used as the tracking target. Subsequently, migratory values were statistically analyzed across experimental conditions. Data are presented as mean ± SEM and statistical significance was assessed by using the Student paired t test, in which p<0.05 was defined as statistically significant.

### Cell death analysis by time-lapse

Time-lapse videos obtained as described above were also used to asses cell death by necrosis and apoptosis in control and 0G treated mixed glial culture of GFP labeled OPCs. Necrotic and apoptotic OPCs were manually identified in video-image sequences by tracing morphological changes associated with necrosis and apoptosis in individual cells. OPCs showing morphological alterations such as plasma membrane blebbing, loss of processes, nucleus karyolysis/pyknosis and chromatine condensation were included in the analysis.

## Results

### The impact of microgravity on the proliferation of mouse OPCs

**Figure 1:**
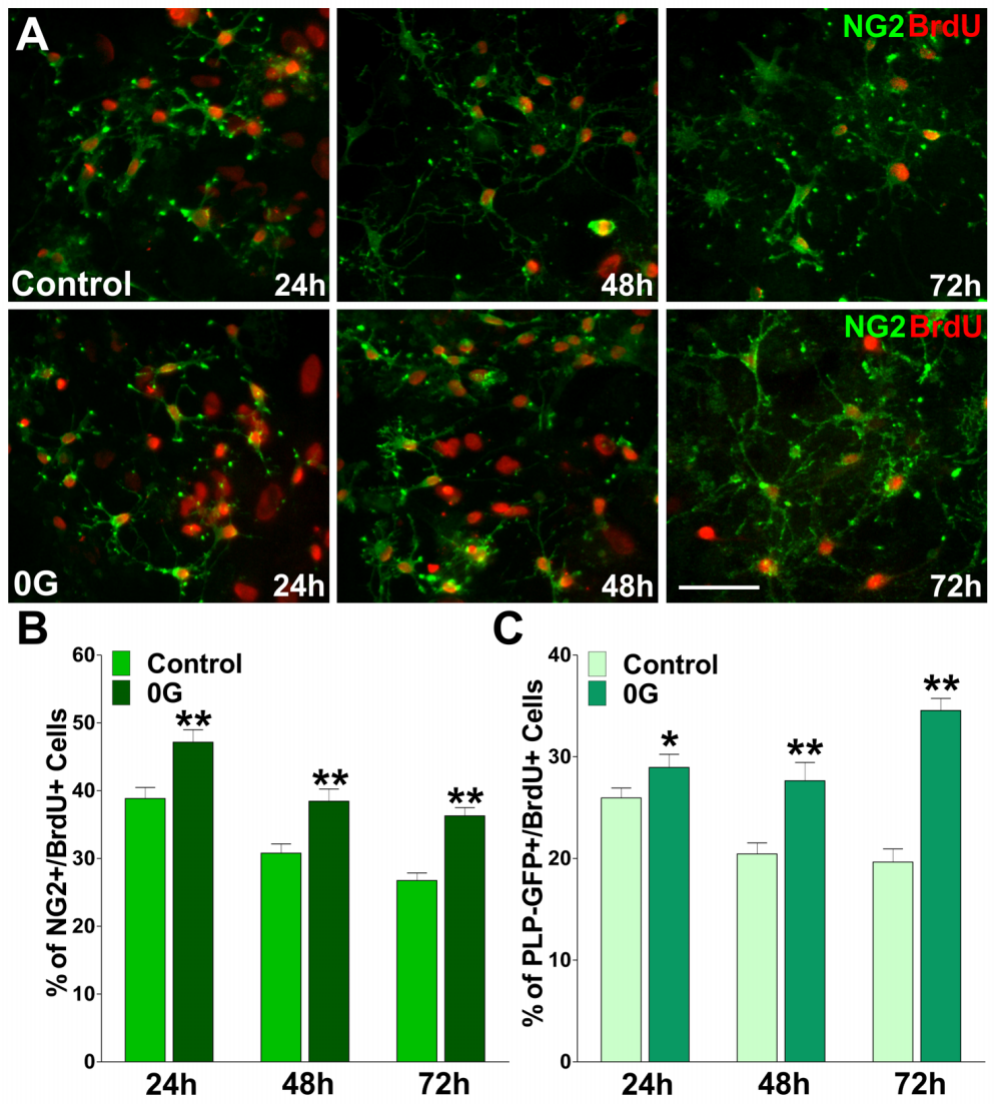
Proliferation of PLP-GFP-expressing OPCs is increased by simulated microgravity. Mixed glial cultures of PLP-GFP labeled OPCs were treated during 24, 48 and 72 h in 0G. Twenty-four hour pulses of 10 µM bromo-deoxyuridine (BrdU) were begun at 0 h, 24 h and 48 h. After each BrdU pulse, cells were fixed and immunostained with anti-BrdU and anti-NG2 antibodies. (**A**) Microphotographs showing NG2+/BrdU+ cells at 24, 48 and 72 h. Green: NG2 immunostaining, and Red: BrdU immunostaining. Scale bar = 50 µm. (**B and C**) The percentage of NG2+/BrdU+ and PLP-GFP+/BrdU+ cells in each experimental condition was compared with respective controls. Values are expressed as mean ± SEM of two independent experiments. *p<0.05, **p<0.01 versus respective control.

We have performed experiments to assess the effect of microgravity on OPC proliferation. The OPCs used in these experiments were obtained from transgenic mice expressing GFP under control of the PLP promoter [11]. In these mice, GFP expression provides a convenient marker for OPCs in the imaging experiments in mixed glial cultures. Initially, we subjected mouse mixed glial culture of GFP labeled OPCs to a short exposure of simulated microgravity (**0G**) produced by a 3D-Clinostat robot. Since actively proliferating cells duplicate their DNA content, we labeled proliferating OPCs from control and 0G treated cultures with the thymidine analogue bromo-deoxyuridine (BrdU) over a period of three days. Twenty four hour pulses of 10 µM BrdU were given at 0 h (immediately before starting the 0G treatment), 24 h and 48 h (**Figure 1A**). After each BrdU pulse, proliferating progenitors were identified by double immunofluorescence for BrdU and NG2 and the relative number of NG2^+^/BrdU^+^ cells was quantified in each cell population (**Figure 1B**). We found that cell proliferation was significant higher in the 0G treated populations than control cultures (**Figure 1B**). For example, after 24 h the average number of proliferating cells in the 0G treated culture (47%) was significantly higher than that of the control group (39%, P<0.01) (**Figure 1B**). Similar results were found after 48 and 72 h of 0G treatment (**Figure 1B**). Additionally, we evaluated the total number of PLP-GFP^+^/BrdU^+^ cells in both experimental groups. In agreement with our previous findings, 0G treatment was able to significantly increase the total number of PLP-GFP^+^/BrdU^+^ cells (**Figure 1C**).

**Figure 2:**
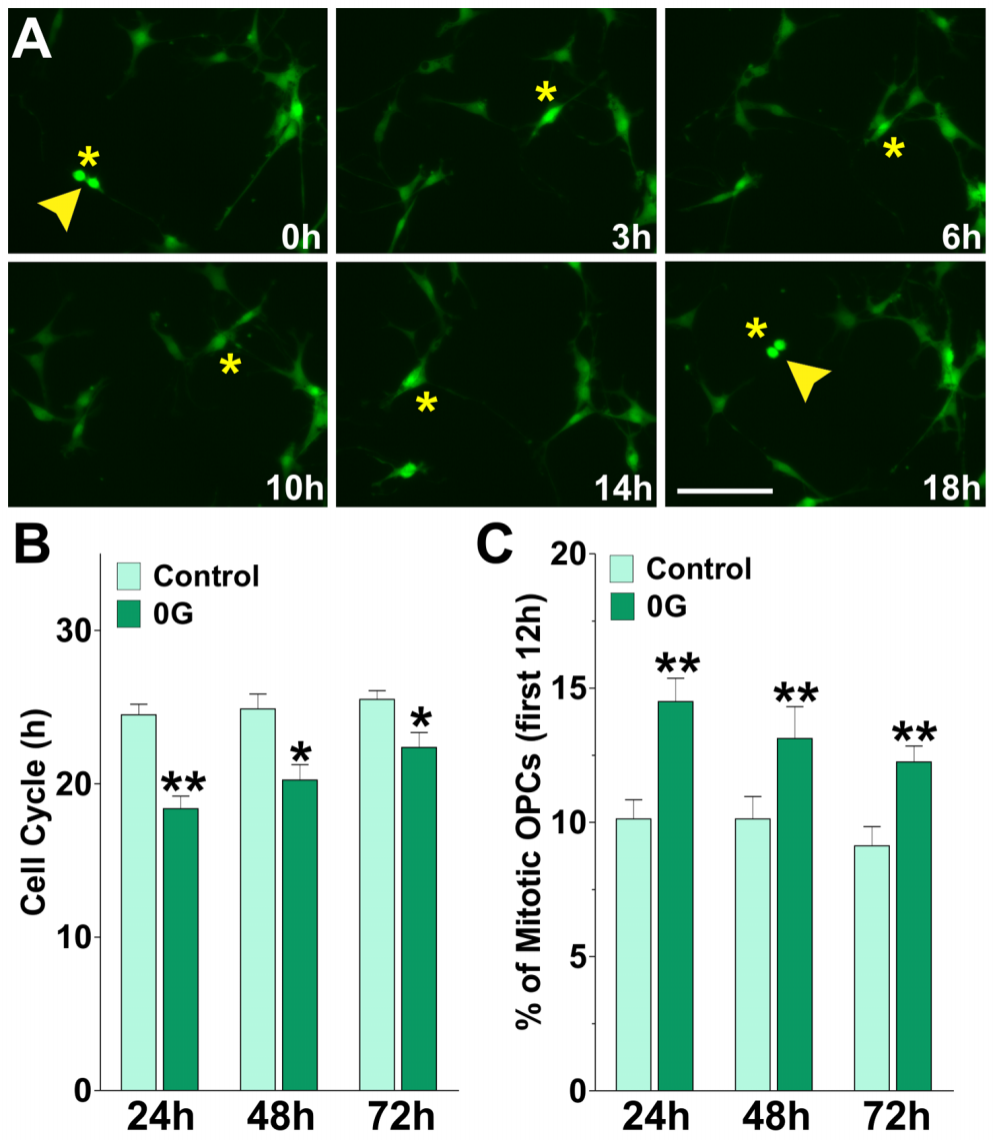
Cell cycle shortening in microgravity. Mixed glial cultures of PLP-GFP labeled OPCs were incubated in a chamber with 5% CO2 at 37°C, which was placed on the stage of a spinning disc confocal microscope. GFP-labeled OPC clones were imaged with a specific GFP filter at 6 min intervals for a period of 26 h. (**A**) Time-lapse series of OPC∼GFP clones from cultures treated during 24 h in 0G. Yellow arrowheads designate cytokinesis events. Tracking of cells between birth cytokinesis and division cytokinesis was noted with a yellow asterisk near the cell, which was generated from frame to frame. Each frame represents a single section of a time lapse video sequence. Time is denoted in hours in the bottom right corner. Scale bar = 50 µm. (**B and C**) Estimated cell cycle times and percentage of mitotic OPCs for each experimental condition. Values are expressed as mean ± SEM of four independent experiments. *p<0.05, **p<0.01, versus respective control.

Next, we measured the cell cycle times of treated (0G) and control (1G) cells by performing time lapse imaging in mixed glial cultures using GFP labeled OPCs. We imaged individual clones of GFP-labeled OPCs (OPC∼GFP) in the presence of an astrocyte monolayer, a known source of growth factors such as PDGF, bFGF and IGF-1 [13]. These experiments were performed for a period of 26 h. In these time-lapse experiments, cell proliferation was assessed by calculating the average cell cycle time in different OPC∼GFP clones. Cell cycle time was defined as the period between when a cell was first generated by cytokinesis and when that cell subsequently divided, giving birth to two daughter cells. The time at which cytokinesis occurred was considered to be the first appearance of a distinct border between two daughter cells in videomicroscopic image sequence [14]. Examples of cytokinetic events in GFP-labeled OPCs grown for 24 h on 0G are shown in **Figure 2A**. 120 cytokinetic events were randomly selected from movies at different time points. Tracking of cells was performed by visual observation of image sequences as described previously [14]. In some cases the cells were semi-automatically followed with the SlideBook program by attaching a number to the cell, which was propagated from frame to frame. Tracking was performed forwards and backwards in time from each identified cytokinesis events to maximize the number of lineally related cytokineses identified. Furthermore, the percentage of cycling OPCs during the first 12 h of the time-lapse experiment was performed by visual observation. Control OPCs showed a Tc of ∼24 h, whereas the cell cycle time of OPCs subject to microgravity was decreased to ∼18 h, ∼20 h and ∼22 h after 1, 2 and 3 days in 0G respectively (**Figure 2B**). Furthermore, as shown in (**Figure 2C**), the percentage of cycling OPCs during the first 12 h of the time-lapse experiment was significantly higher in the 0G treated cultures suggesting that the fraction of proliferating OPCs is increased by simulated microgravity. These results indicated by several independent measures that microgravity promoted OPC proliferation, i.e. an increase in the number of mitotic cells and a decrease in the duration of the cell cycle.

### Assessment of the effect of 0G on mouse oligodendrocyte lineage progression

**Figure 3:**
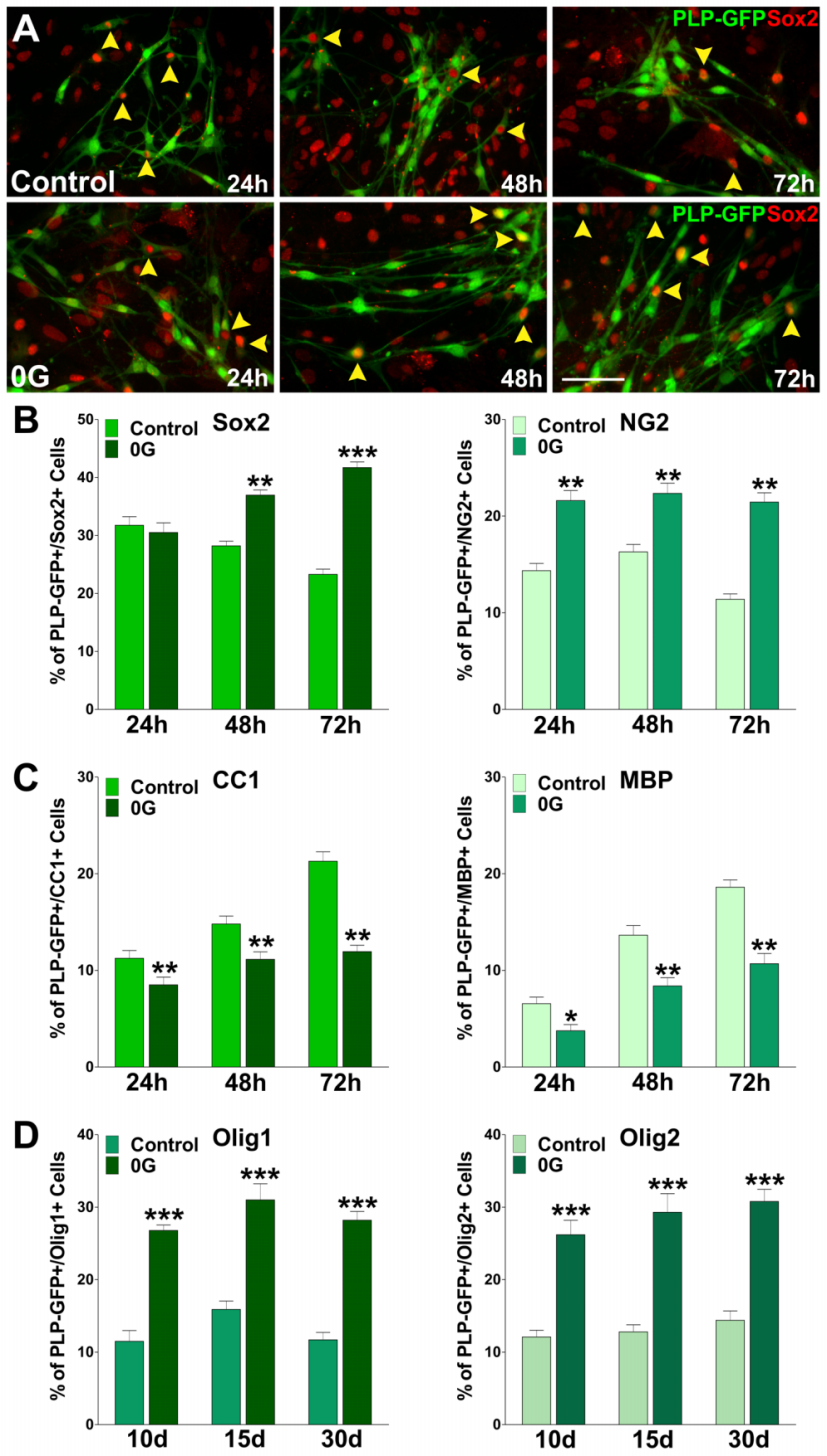
Microgravity increases OPC numbers and oligodendrocytes remained immature. Mixed glial cultures of PLP-GFP labeled OPCs were treated during 24, 48 and 72 h in 0G. (**A**) Yellow arrowheads define PLP-GFP/Sox2 double positive cells at 24, 48 and 72 h. Green: PLP-GFP, Red: Sox2 immunostaining. Scale bar = 50 µm. (**B and C**) After treatment, cells were fixed and immunostained for several oligodendrocyte stage markers and the percentage of positive cells in each experimental condition was analyzed by confocal microscopy. (**D**) Mixed glial cultures of PLP-GFP labeled OPCs were treated during 10, 15 and 30 days in 0G. After treatment, cells were fixed and immunostained for Olig1 and Olig2 and the percentage of positive cells in each experimental condition was analyzed by confocal microscopy. Results are the means ± SEM for three independent experiments. *p<0,05, **p<0,01 and ***p<0,001 vs. respective controls.

The results described above clearly indicate that simulated microgravity modulates OPC proliferation. Since cell cycle exit is a prerequisite for OPC lineage progression [15], we further investigated the effect of 0G on OPC differentiation. The effect of simulated microgravity on OPC maturation was tested in enriched cultures of PLP-GFP mouse oligodendrocytes. Our data showed that the expression of mature oligodendrocyte markers was significantly delayed in 0G treated OPCs as shown in **Figure 3C**. Under conditions where OPCs were allowed to differentiate for 72 h, a decrease in the proportion of cells that expressed mature specific markers such as CC1 and MBP was induced by simulated microgravity (**Figure 3C**). On the other hand, 0G treated cultures showed an increased number of cells that retained immature oligodendrocyte markers such as Sox2 and NG2 (**Figure 3A and B**). Similar results were found after 10, 15 and 30 days of microgravity treatment (**Figure 3D**). These long-term 0G cultures display a significant surge in the number of Olig1 and Olig2 positive OPCs and demonstrate that long periods of 0G exposure are not deleterious for OPCs. Importantly, the number of Sox2 positive cells was similar to control levels during the first 24 h of the 0G treatment (**Figure 3A and B**), suggesting that this particular OPC population are less susceptible to micrograbity than NG2 positive cells.

Next, we measured necrotic and apoptotic cell death of treated (0G) and control (1G) mixed glial cultures by time lapse microscopy. After 24 h in the 3-D Clinostat, GFP labeled OPCs were placed on the stage of a spinning disc confocal microscope for an additional 24 h. After collecting the videos, cell death was assessed by counting the total number of necrotic and apoptotic OPCs. Necrotic and apoptotic cells were identified by changes in cell morphology such as plasma membrane blebbing, loss of processes, nucleus karyolysis/pyknosis and chromatine condensation. We found that OPCs cultured in microgravity for 24 h showed no increase in the percentage of necrotic/apoptotic cells relative to control. Only 1,7% of control and 1,8% of 0G treated OPCs die by necrosis or apoptosis during the first 12 h of this time-lapse experiment (p = 0.42).

### Microgravity enhances mouse OPCs migration

**Figure 4:**
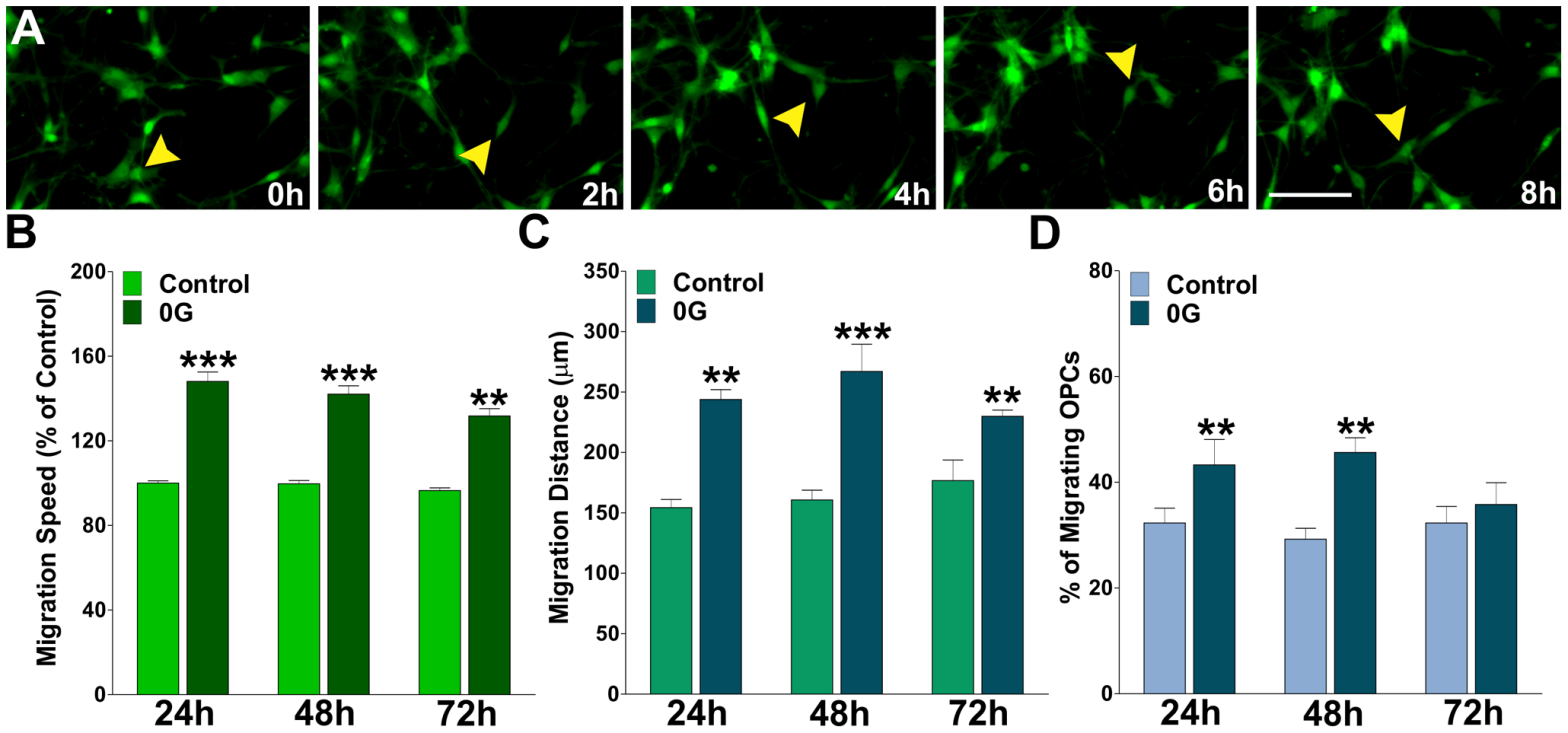
Simulated microgravity increases OPC migration. Mixed glial cultures of PLP-GFP labeled OPCs were imaged with a GFP filter at 6 min intervals for a period of 16 h. (**A**) Time-lapse series of OPCs from cultures treated during 24 h in 0G. Yellow arrowheads identify a migrating OPC. Each frame represents a single section of a time lapse video sequence. Time is denoted in hours in the bottom right corner. Scale bar = 50 µm. Cell migration speed and distances were analyzed off-line by tracing individual cells at different times, after which migratory values were statistically analyzed across the experimental conditions. (**B**) OPC migration speed was calculated from at least 60 cells in each experimental condition. (**C and D**) Total migration distance was followed for 12 h in 50 cells from each experimental condition and the percentage of migrating cells was calculated from the entire cell population. Values are expressed as mean ± SEM of at least four independent experiments. **p<0.01, ***p<0.001 versus control cells.

Using time-lapse video microscopy we examined the effect of simulated microgravity on OPC migration. These experiments were performed over a period of 72 h on mouse mixed glial culture of GFP labeled OPCs. In this time-lapse two-dimensional cell migration assay, cell movement was assessed by calculating the average cell migration velocity and the total distance traveled by the cell. For this analysis, only GFP positive OPCs moving more than 50 µm in 6 h were scored. Immediately after removal from the 3-D Clinostat OPCs were placed on the stage of a spinning disc confocal inverted microscope and tracked using the SlideBookTM 4.1 data analysis program [12]. Migrating OPCs were automatically followed by tagging a color or number to each cell examined, which were then tracked from frame to frame. Examples of such measurements are shown in **Figure 4A**. Under these experimental conditions the mean rate of migration for control and 0G treated OPCs during the first 24 h was 12±3 µm/h and 21±2 µm/h, respectively, P<0.001 (**Figure 4B**). Thus, the average cell migration velocity in 0G treated OPCs was significantly higher compared with that of the control group (1G). Consequently, there was an increase in the total migration distance (**Figure 4C**), as well as, in the number of migrating cells in the 0G OPC population (**Figure 4D**).

**Figure 5:**
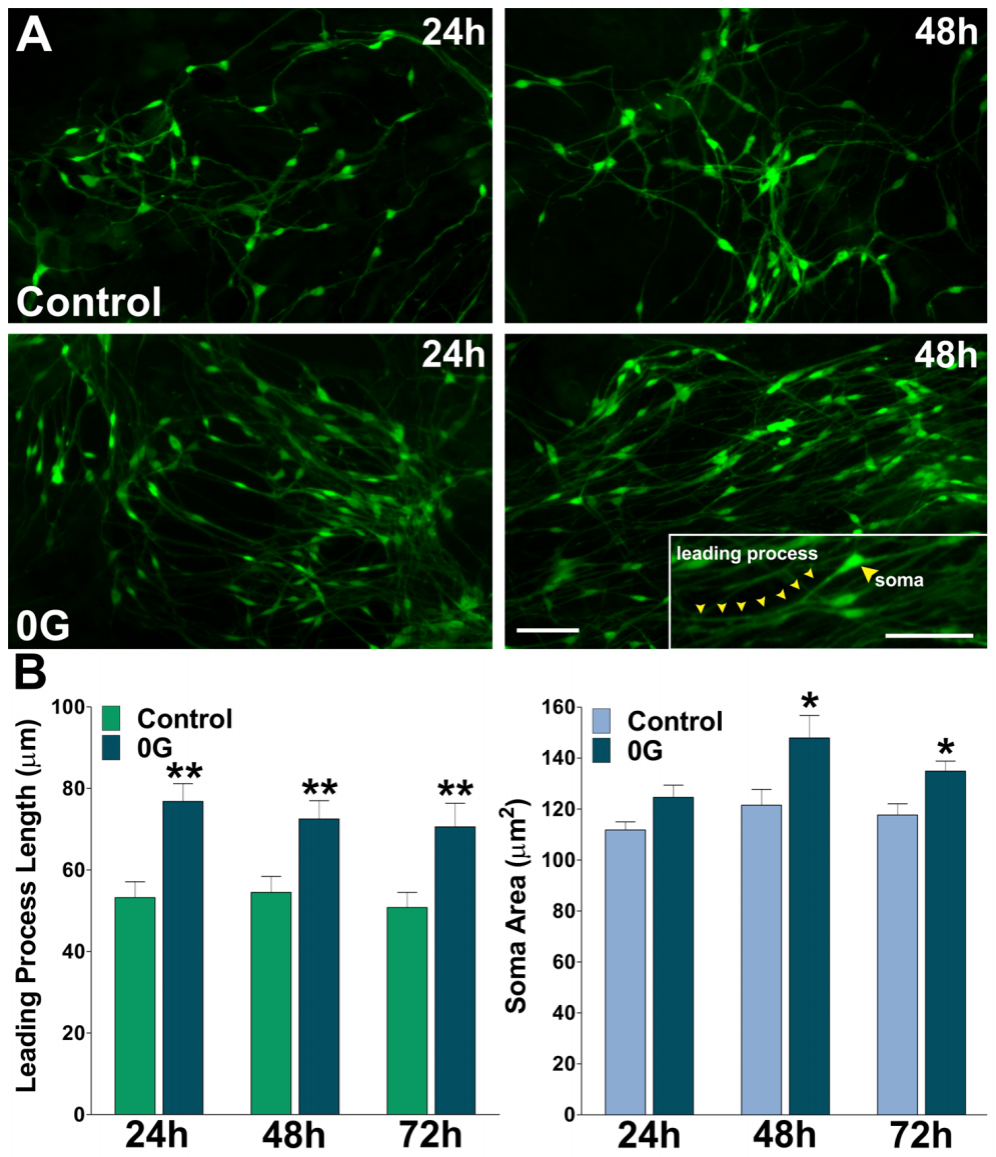
Simulated microgravity promotes the extension of the leading process in OPCs. (**A**) Mixed glial cultures of PLP-GFP labeled OPCs were incubated in a chamber with 5% CO2 at 37°C, which was placed on the stage of a spinning disc confocal microscope. Pictures show examples of living GFP-labeled OPCs that were imaged for a period of 26 h. Scale bar = 50 µm. In the insert, a single migrating OPC is shown, small yellow arrowheads point at the leading process and the cell soma is shown by the large arrowhead. Scale bar = 50 µm (**B**) The average leading process length and soma area of migrating OPCs was calculated from at least 50 cells in each experimental condition. Values are expressed as mean ± SEM of at least four independent experiments. *p<0.05, **p<0.01 versus control cells.

Migrating cells move in a saltatory fashion, alternating periods of higher and lower speed at a frequency of ∼1–2 cycles/h. These cycles reflect the steps required for directed OPC movement: extension of the leading process, translocation of the soma/nucleus (nucleokinesis), and retraction of the trailing process, these three individual steps constitute a single migration cycle [12]. Using high resolution spatiotemporal microscopy, we determined the average length of individual leading processes in migrating OPCs before the initiation of the migration cycle (before nucleokinesis). We found that the average leading process was significant longer in 0G treated OPCs than in corresponding control cells (**Figure 5A and B**), demonstrating that during OPC migration simulated microgravity promotes leading process extension. Taken together these experiments localized the step in the migration process in which simulated microgravity plays a role and suggest that 0G modulates OPC migration by accelerating both nucleokinesis and leading process growth. Additionally, and consistently with highly proliferative cells, we also found an enlargement of the cell soma volume in OPCs incubated under simulated microgravity for two and three days (**Figure 5B**).

### Impact of 0G on the cell cycle of human OPCs derived from neural stem cells

**Figure 6:**
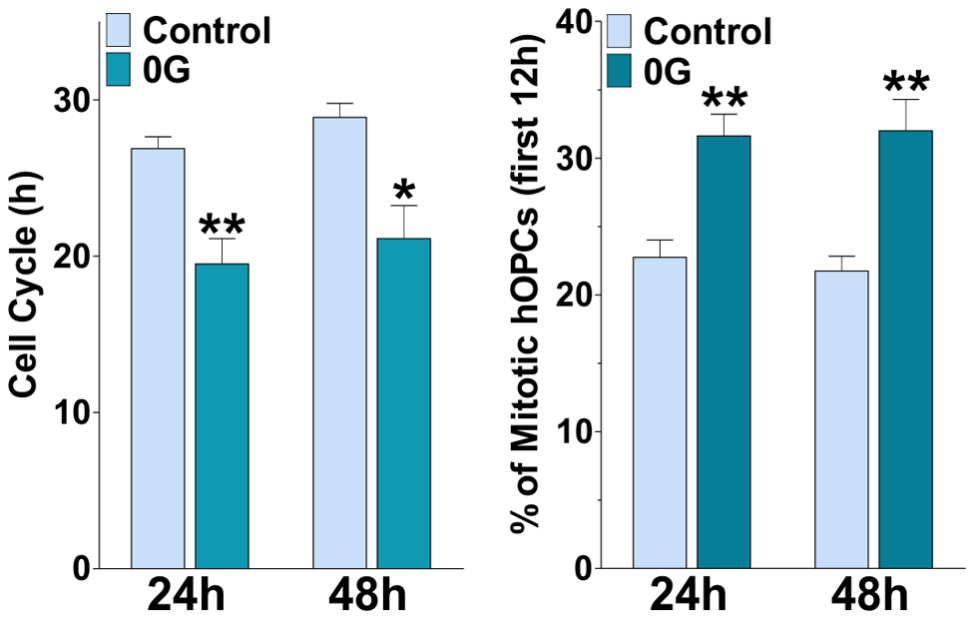
Like in mouse OPCs simulated microgravity shortens the cell cycle of human OPCs (hOPCs). hOPCs were incubated in a chamber with 5% CO2 at 37°C, which was placed on the stage of a spinning disc confocal inverted microscope. Cells were imaged at 6 min intervals for a period of 26 h. Estimated cell cycle times and percentage of mitotic hOPCs for control and 0G treated cultures are shown. Values are expressed as mean ± SEM of four independent experiments. *p<0.05, **p<0.01, versus respective control.

In order to assess if the effects of simulated microgravity on cell proliferation OPCs were an indirect effect through the presence of astrocytes and their secreted factors, we prepared pure OPCs cultures derived from human neural stem cells isolated from the brain of human embryos to assess the effect of microgravity on pure populations of human OPCs (**hOPCs**) in the absence of astrocytes. These cultures were prepared as previously described [9], [7] using our proprietary culture medium specifically designed for OPCs [9]. We measured the cell cycle duration of treated (0G) and control (1G) cells by performing time lapse microscopy on these cultures. We imaged individual clones of human OPCs, in which at difference with the PLP-GFP mouse OPCs that were cultured in the presence of an astrocyte monolayer, the source of growth factors such as PDGF and bFGF that astrocytes synthetize [13], was absent. Interestingly, the number of mitotic figures increased in cultures maintained in 0G when compared with their 1G counterpart (**Figure 6**). The cell cycle of human OPCs kept in 0G was also shortened (**Figure 6**). Therefore, our results confirm that simulated microgravity increases cell proliferation and reduces the length of the cell cycle in human OPCs in a direct manner in the absence of astrocytes (**Figure 6**).

**Figure 7:**
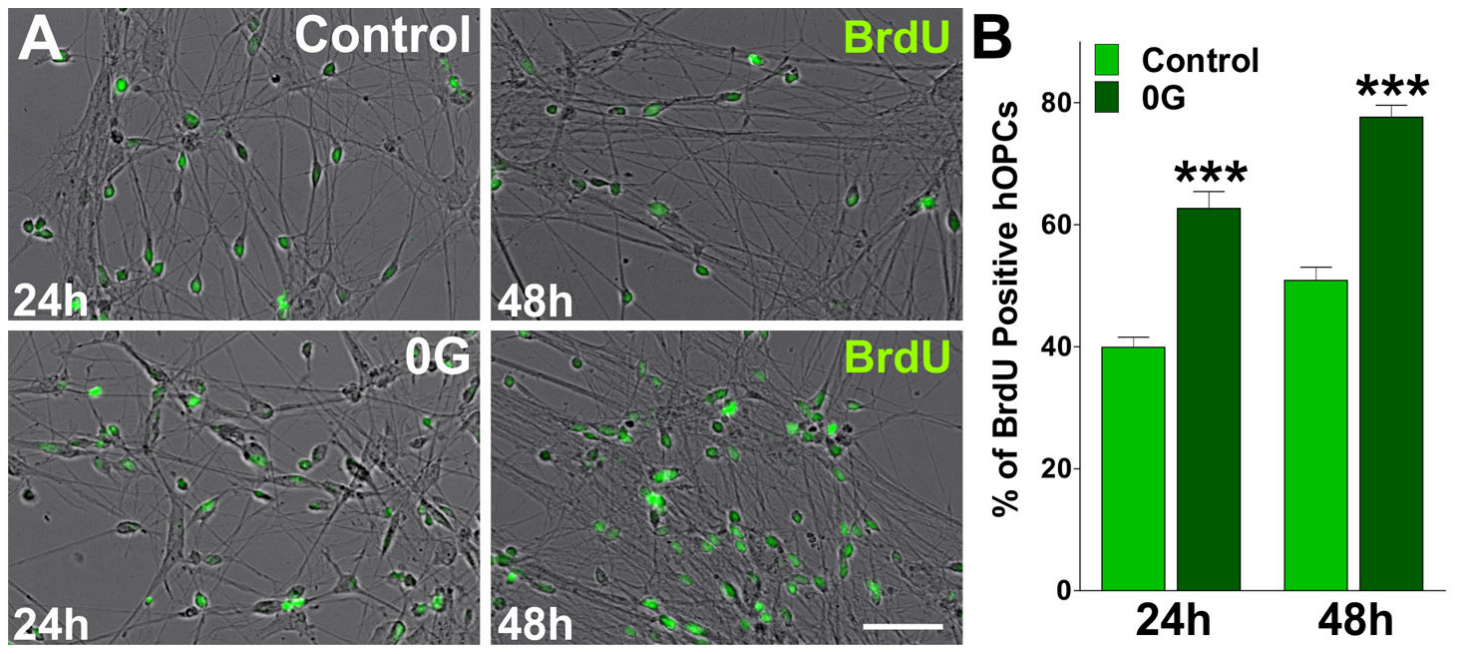
Simulated microgravity increases the number of DNA-synthesizing hOPCs. Cultures of hOPCs were treated during 24 and 48-four hour pulses of 10 µM bromo-deoxyuridine (BrdU) were done during the 0G treatment as well as in parallel untreated cultures. After each BrdU pulse, cells were fixed and immunostained with anti-BrdU. (**A**) Microphotographs showing BrdU positive cells at 24 and 48 h. green: BrdU immunostaining. Scale bar = 50 µm. (**B**) The percentage of BrdU positive cells in each experimental condition was compared with respective controls. Values are expressed as mean ± SEM of two independent experiments. ***p<0.001 versus control.

We next used BrdU over a period of two days to label proliferating human OPCs from control and 0G treated cultures. Twenty four hour pulses of 10 µM BrdU were given at 0 h, (immediately before starting the 0G exposure), 24 h and 48 h. After each BrdU pulse, proliferating progenitors were identified by immunofluorescence for BrdU (**Figure 7A**). We found that cell proliferation was significant higher in the 0G treated populations than in control cultures. For example, after 24 h of treatment the average number of proliferating cells in the 0G treated cultures was ∼20% higher than that in the control group (**Figure 7B**). Furthermore, after 48 h of exposure to 0G there was ∼28% more BrdU positive cells than in cultures maintained in 1G (**Figure 7B**). In agreement with our previous findings in mouse mixed glial cultures, 0G treatment was able to significantly increase the total number of mitotic human OPCs.

### Simulated microgravity enhances human OPCs motility

**Figure 8:**
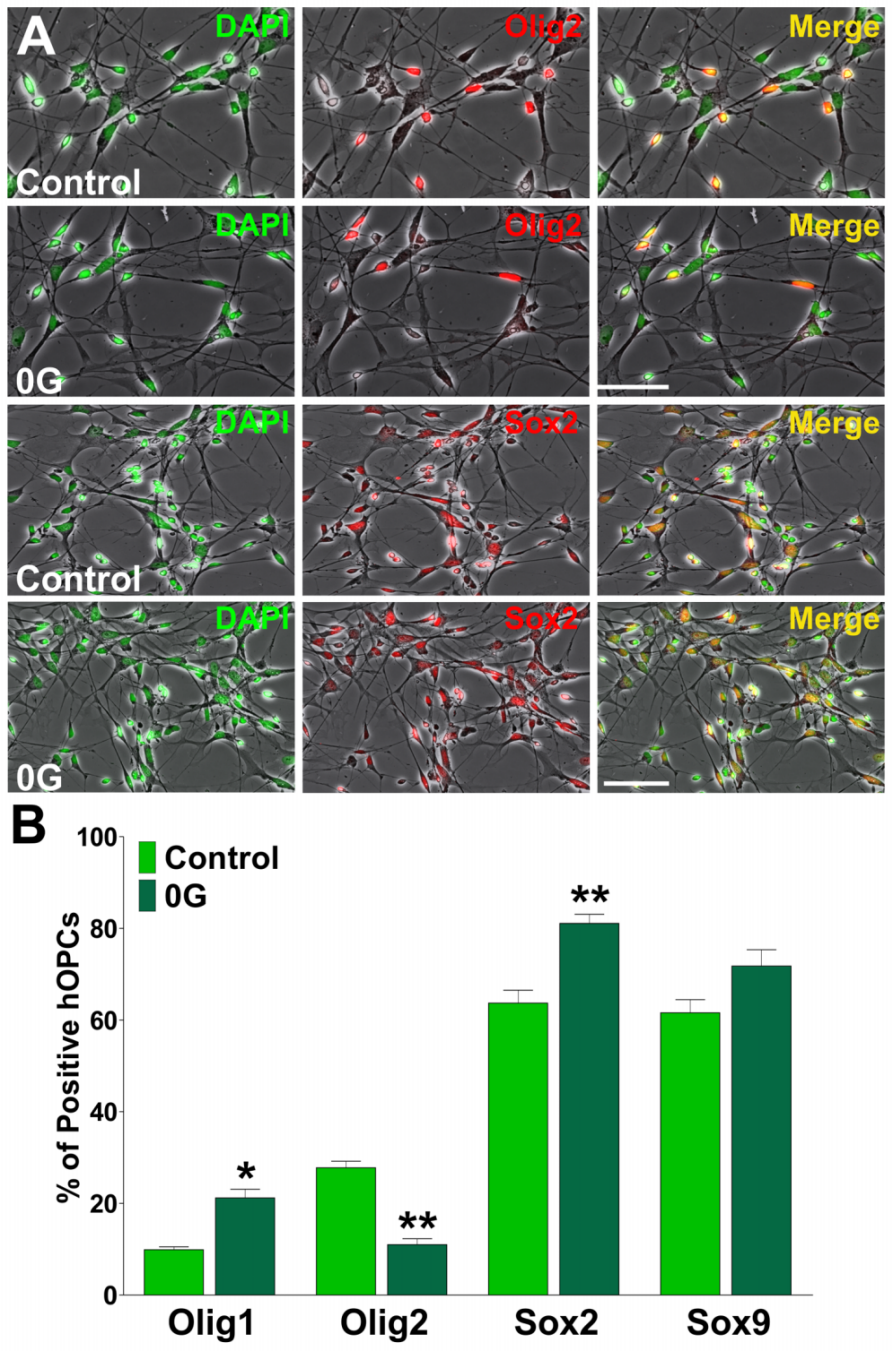
Simulated microgravity maintains the expression of early developmental markers in hOPCs. Cultures of hOPCs were treated during 24(**A**) Microphotographs showing Olig2 and Sox2 positive cells in control and 0G treated cultures. Scale bar = 50 µm. (**B**) The percentage of positive cells in each experimental condition was analyzed by confocal microscopy. Results are the means ± SEM for three independent experiments. *p<0.05, **p<0.01, versus respective control.

**Figure 9:**
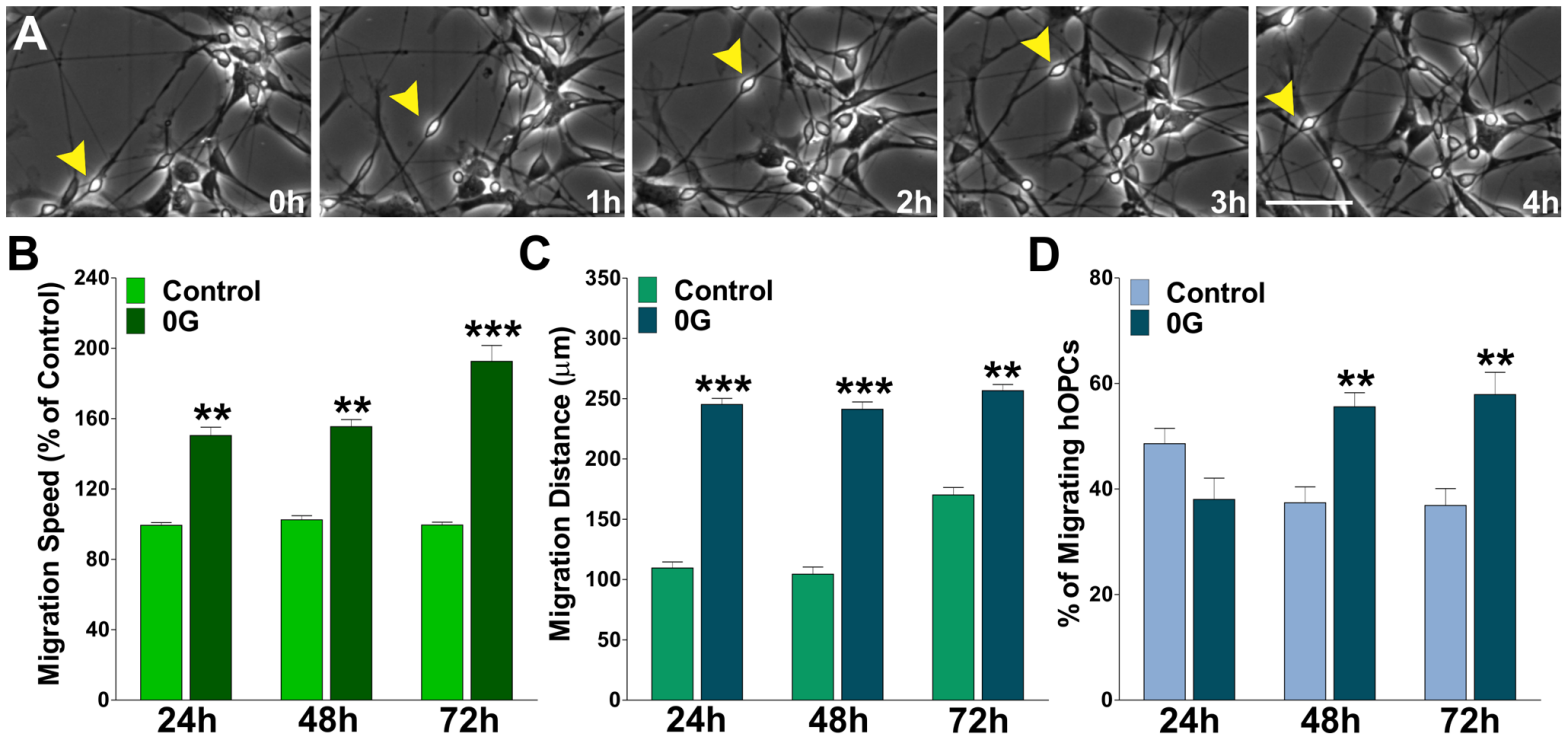
Simulated microgravity enhances hOPCs migration. hOPCs were imaged at 6(**A**) Time-lapse series of hOPCs from cultures treated during 24 h in 0G. Yellow arrowheads identify a migrating hOPC. Each frame represents a single section of a time lapse video sequence. Time is denoted in hours in the bottom right corner. Scale bar = 50 µm. Cell migration speed and distances were analyzed off-line by tracing individual cells at different times, after which migratory values were statistically analyzed across the experimental conditions. (**B**) hOPC migration speed was calculated from at least 60 cells in each experimental condition. (**C and D**) Total migration distance was followed for 12 h in 50 cells from each experimental condition and the percentage of migrating cells was calculated from the entire cell population. Values are expressed as mean ± SEM of at least four independent experiments. **p<0.01, ***p<0.001 versus control cells.

Human OPCs cultured under simulated microgravity as well as those cultured under 1G conditions expressed Olig1, Olig2, Sox2 and Sox9 (**Figure 8**). We didn't find any significant difference in the expression of Sox9 but we found a significant reduction in the expression of Olig2 and a relative increase in the number of Olig1 and Sox2 positive hOPCs under simulated microgravity (**Figure 8B**). Therefore, human OPCs cultured in simulated microgravity were considered to be in a greater undifferentiated state that those grown in 1G and consequently to possess high migration ability. In agreement with our previous data in mouse mixed glial cultures, human OPCs cultured under microgravity exhibited greater migratory values compared with cells cultured in 1G (**Figure 9**). OPCs cultured under microgravity were found to have greater average migration speed (**Figure 9B**), larger total migration distance (**Figure 9C**), and more migratory cells (cells moving more than 50 µm in 6 h) (**Figure 9D**).

## Discussion

During the past four decades our lab has investigated oligodendrocyte development in health and disease contributing to a better knowledge of these cells [5], [9], [16]–[18]. We have previously established protocols to manipulate the commitment, proliferation and progression from the OPC stage to a mature and functional myelinating stage [8]–[9]. The present study is the first to introduce simulated microgravity as a novel approach to increase the proliferation of neural cells and in particular OPCs aiming at increasing their numbers for cell replacement therapies in regenerative medicine. The developmental principles known to occur in rodent and human oligodendrocytes *in vivo* have been well documented and they also occur closely when these cells are in culture [19]. Here we wanted to ascertain that these developmental milestones still occur in OPCs exposed to simulated microgravity. Consequently, we examined three fundamental aspects in the biology of OPCs: proliferation, migration and cell differentiation.

### Simulated microgravity stimulates OPC proliferation

We found simulated microgravity to be an excellent strategy to increase cell numbers without performing genetic manipulation or long term treatments with mitogens. The primary goal of this study was to determine if cells remained viable and functional after exposure to 0G rather than the characterization of the mechanisms or molecules leading to cell proliferation. Nonetheless, it is important to note that numerous studies have reported that OPCs remain immature and continue to proliferate upon continuous exposure to mitogens and they move forward towards mature oligodendrocytes in response to mitogen withdrawal [20]–[23]. The present study used OPC enriched mix glial cultures derived from postnatal mouse brain cultured in a specific medium as previously described without adding any mitogens (i.e. bFGF or PDGF) with the sole difference being exposure or non-exposure to simulated microgravity. One could hypothesize that OPC mitogens are produced and secreted by astrocytes in higher levels when exposed to 0G, contributing at least in part to the increase in OPCs proliferation. Nonetheless, our data obtained using pure human OPC cultures demonstrate that cell proliferation is increased by simulated microgravity in spite of the absence of astrocytes and the mitogens they produce. Moreover, the cell cycle was shortened in human OPCs grown in 0G suggesting that the physical dynamics conferred to these cells by simulated microgravity may play a role on the cell cycle changes that we observed.

The cytoskeleton forms the main structural component of cells and consists of interactions between microtubules, microfilaments, intermediate filaments and associated proteins [24]. The cell cytoskeleton appears to be related to cell shape and architecture [25]. Changes in cell architecture will impact the way cells respond to their environment and vice versa, the environment may induce cytoskeleton changes [26]. Alterations in the cyto-architecture can be brought about by changes in the way microtubules and other cytoskeletal components behave in microgravity [26]–[27]. Karyokinesis and cytokinesis are cellular processes involved in spindle formation and cell membrane constriction during cell division and both processes depend on normal microtubule polymerization [29], [43]. Therefore, we could infer that increased OPC proliferation in simulated microgravity may be explained by cytoskeleton changes that accelerate karyokinesis and cytokinesis under microgravity conditions.

### Simulated microgravity promotes OPC migration

During development, postmitotic cells of the CNS including neurons and myelin-forming oligodendrocytes migrate from the germinal areas of the neural tube to their final destination sites [30]–[32]. The cellular and molecular bases of OPCs migration during development have been established [33]. Moreover, the innate and extensive migratory capacity of OPCs is consistent *in vivo* during development and after transplantation into the post-natal and the adult CNS of myelin deficient animal models [19], [34]–[37].

Migration of cells due to cytoskeletal changes is a very important physiological phenomenon which if disturbed can lead to several detrimental effects on the body. Gravitational forces may be experienced by individual cells in the living organism as a result of stress-dependent changes in cell, tissue, or organ structure that, in turn, alter extracellular matrix mechanics, cell shape and cytoskeletal organization [38]. Cytoskeletal components such as microtubules are changed in microgravity, and this is presumed to explain some of the effects of microgravity on cells [39]. For instance, Siamwala and coworkers [40] showed that simulated microgravity perturbs actin polymerization and promote nitric oxide-associated migration in a human immortalized cell line.

Our data indicate that microgravity plays a role in the migration of oligodendrocytes progenitor cells. We have examined the migration ability of OPCs maintained in 1G vs. those kept in simulated microgravity. Two parameters of cell migration were examined: migration speed and migration distance. Immediately after removal from the 3-D Clinostat cells were placed in our microscope time-lapse system. We found that cells exposed to simulated microgravity migrated faster than sister cultures maintained in 1G. Further analysis showed that 0G exposed OPCs migrate longer distances than those kept in 1G. Our data also demonstrated that more cells were migratory in cultures exposed to 0G when compared to cells cultured in 1G. Together, our data indicate a profound effect of simulated microgravity on OPC motility. Since migration of oligodendrocyte progenitor cells from proliferative zones to their final location in the brain is an essential step in nervous system development and repair, OPC expanded under simulated microgravity is an exceptional and ideal cell population for cell replacement therapies where grafted cells should migrate to reach naked axons in the host CNS.

### Oligodendrocyte maturation is delayed in 0G

It has been reported that simulated microgravity suppresses the differentiation of human osteoblast cells [2], human hematopoietic progenitor cells [1] and rat myoblasts [2]. Yuge et al. [2] showed that human mesenchymal stem cells cultured under simulated microgravity maintained their undifferentiated state. In the same line, our results showed a significant delay of cell maturation in mouse OPCs that was further confirmed in cultures of human oligodendrocytes free of astrocytes and neurons. Therefore, it is neither a growth factor(s) neither a cell dependent phenomenon. It has been proposed that cell cycle exit is a prerequisite for oligodendrocyte differentiation [15] and simulated microgravity is actively promoting OPC division and motility, this apparent lack of cell maturation could be a secondary effect. Moreover, human OPCs were maintained in OSM that is designed to support the needs of the OPC stage rather than lineage progression [9]. Alternatively, lack of maturation of OPCs while in 0G could be a direct effect of microgravity on signaling that may modulate the differentiation program. To this effect, the role of the oligodendrocyte cytoskeleton in OPC differentiation and myelination has been described in a recent review [41]. During the first stage of myelination, oligodendrocyte processes are extended and branched. The formation and extension of processes in oligodendrocytes, as in all other cell types, require the thorough reorganization of the cytoskeleton with the formation of filopodia and lamellipodia mediated by actin polymerization, and the subsequent invasion of microtubules [41]. Additionally, myelin-specific proteins, such as PLP and MAG, are transported in a microtubule-based manner through the Golgi to the growing myelin sheet, where they are incorporated into the membrane, resulting in further membrane specialization and maturation of the insulating myelin sheath [28], [42]. Consequently, processes formation and branching is a prerequisite for OPC maturation. Moreover, considering that the developmental length for human oligodendrocytes *in vivo* is much longer than in mouse we could interpret the delayed maturation data from the human OPC study to be a combination of the various possibilities mentioned above. Since microgravity affects microtubules and microfilaments polymerization [27] further experiments will be needed to establish a possible direct effect of microgravity on oligodendrocyte morphological maturation and subsequently on myelin synthesis.

In this report we have established that proliferation and migration are part of the broad plasticity of the oligodendrocyte progenitor cells that can be modulated by simulated microgravity. Basically, we have revealed that simulated microgravity promotes mouse and human OPC proliferation and motility maintaining these cells in an immature stage. Since effective methods to generate OPCs for cell replacement therapies are still needed, we believe that the present study provides a novel platform opening new fields of research using gravitational science for regenerative medicine, particularly to address neurodegenerative and developmental disabilities associated with myelin loss.
